# Genome-Wide Analysis of GmMYB S20 Transcription Factors Reveals Their Critical Role in Soybean Nodulation

**DOI:** 10.3390/plants14142240

**Published:** 2025-07-20

**Authors:** Junchen Leng, Ruobing Xu, Yanshuang Liu, Tianshu Jiang, Haiying Hu, Zhaojun Ding, Shaojun Dai

**Affiliations:** 1 Development Center of Plant Germplasm Resources, College of Life Sciences, Shanghai Normal University, Shanghai 200234, China; lengjc1@163.com (J.L.); yanshuangliu@yeah.net (Y.L.); 2 School of Life Sciences, Shandong University, Qingdao 266228, China; lcu_xuruobing@163.com (R.X.); 13153305835@163.com (T.J.); 19819748098@163.com (H.H.); dingzhaojun@sdu.edu.cn (Z.D.)

**Keywords:** *GmMYB62a* and *GmMYB62b*, transcription factor, soybean, nodulation

## Abstract

Soybean relies on symbiotic nitrogen fixation (SNF) to support sustainable agriculture. In this study, we conducted a comprehensive analysis of the GmMYB transcription factor subfamily 20, with a focus on *GmMYB62a* and *GmMYB62b*. Phylogenetic and structural analyses revealed that these genes are evolutionarily conserved among legumes and possess distinct domain architectures. Expression profiling and GUS staining showed that *GmMYB62a* and *GmMYB62b* are constitutively expressed in nodules. Functional analyses revealed that loss of *GmMYB62s* function significantly reduced nodule density, while overexpression promoted nodulation. Transcriptomic analysis (RNA-seq) further demonstrated that *GmMYB62s* regulate key pathways, including hormone signaling, immune responses, and cell wall metabolism, thereby coordinating symbiotic interactions. Collectively, our findings identify *GmMYB62a* and *GmMYB62b* as critical molecular regulators of nodulation in soybean, providing promising targets for improving symbiotic nitrogen fixation efficiency in legume crops.

## 1. Introduction

Soybean (*Glycine max*) is a globally important economic crop, and its capacity for symbiotic nitrogen fixation (SNF) with rhizobia constitutes a critical pillar of sustainable agriculture [1]. Through SNF, atmospheric nitrogen (N_2_) is converted into plant-assimilable ammonia (NH_3_), thereby reducing dependence on chemical nitrogen fertilizers, enhancing soil fertility, and providing an essential nitrogen source for plant growth [2]. Leguminous crops collectively contribute approximately 21 million tons of biologically fixed nitrogen to agricultural ecosystems each year [3]. However, the global cultivation area of legumes has expanded only modestly over the past five decades [4]. The nitrogen-fixing potential of soybean offers considerable promise for reducing fertilizer inputs, lowering production costs, and alleviating environmental pollution associated with nitrogen runoff [5]. Thus, research on SNF in soybean not only supports the development of high yield, low input, and environmentally sustainable cropping systems but also provides valuable insights into plant microbe symbiotic interactions advancing both agricultural sustainability and ecological resilience [6].

The establishment of symbiotic relationships between legume plants and rhizobia involves a multitude of vital biological mechanisms. During the early stages of infection, plants secrete flavonoid compounds to attract rhizobia. In response, the bacteria attach to the root hair cell walls and secrete Nod factors [7], which trigger oscillatory calcium (Ca^2+^) spiking in the nuclei of root hair cells [8]. This signaling cascade induces localized remodeling of the cell walls, during which pectate lyase (NPL) and the symbiosis-specific pectin methylesterase (SyPME1), acting via the secretory pathway, mediate the degradation of cell wall components. This facilitates plasma membrane invagination and the formation of the initiation site for the infection threads (ITs) [9,10]. In the ITs elongation phase, the “infectosome complex”, composed of exocyst subunit EXO70 family protein H4 (EXO70H4), VAPYRIN (VPY), and LUMPY INFECTIONS (LIN), maintains continuous apical growth of the ITs by regulating polar secretion and vesicle trafficking [11]. Concurrently, symbiosis-specific formin protein SYMBIOTIC FORMIN 1 (SYFO1) mediates the assembly of actin bundles, which drives nuclear migration and guides ITs progression towards the root cortex [12]. Upon entering the nodule organogenesis stage, the cell wall–plasma membrane continuum facilitates the dynamic expansion of the symbiotic interface through vesicle fusion mediated by secretory soluble NSF attachment protein receptor (SNARE) proteins and vesicle-associated membrane proteins 721 (VAMP721) [13,14].

The MYB transcription factors, one of the largest and most functionally diverse transcription factor families in plants, regulate diverse biological processes, including growth, development, metabolism, and stress responses [15]. Based on the number of MYB domain repeats, MYB proteins are classified into four categories: 1R-MYB, R2R3-MYB, 3R-MYB, and 4R-MYB [16]. Among them, the R2R3-MYB subfamily, unique to plants, governs critical processes such as cell proliferation, differentiation, hormone signaling, root architecture, thermotolerance, and responses to abiotic/biotic stresses [17]. Stracke and colleagues first systematically classified the Arabidopsis R2R3-MYB family, revealing its functional sub-functionalization [18]. Subgroup 20 (S20) of the R2R3-MYB family has emerged as a key regulator of plant stress adaptation [15]. In Arabidopsis, AtMYB2 activates abscisic acid (ABA)-dependent drought-responsive genes [19], while its rice homolog OsMYB2 enhances tolerance to salt, cold, and dehydration stress [20]. AtMYB108 (also known as BOS1) mediates resistance to Botrytis cinerea and oxidative stress, and has been recently shown to interact with lateral organ boundaries domain 29 (LBD29) in the regulation of auxin-dependent lateral root development [21]. Homologs from other species, such as TaMYB78 in wheat and CaMYB78 in chickpea, further demonstrate conserved functions in promoting stress tolerance through antioxidant activity and pathogen resistance, respectively [22,23]. Devaiah et al. discovered that AtMYB62 mediates plant adaptive responses to phosphate (Pi) starvation by regulating the gibberellin (GA) biosynthesis pathway, concurrently influencing root development. Phosphorus (Pi) serves as a macronutrient in plants, influencing nodule initiation, development, and N2 fixation. This functional link between Pi signaling/root development mediated by S20 MYB factors like AtMYB62 and the Pi-dependent nature of nodulation suggests a potential role for their homologs in regulating the SNF process [24].

In this study, we identified members of the soybean *GmMYB S20* gene family and conducted comprehensive analyses of their phylogenetic relationships, chromosomal distribution, gene structures, conserved functional domains, and promoter *cis*-regulatory elements. Furthermore, we examined their expression profiles in response to rhizobial symbiosis. Phenotypic characterization of *gmmyb62* and *GmMYB62s* overexpression lines, combined with RNA-seq analysis, revealed that *GmMYB62s* modulates rhizobial symbiosis by regulating hormone signaling, cell wall remodeling, lignin metabolic process, and secondary metabolite biosynthetic process. Collectively, our findings uncover a novel regulatory role for GmMYB62s in legume nodulation and provide a theoretical foundation for the molecular breeding of soybean cultivars with improved nitrogen fixation efficiency.

## 2. Results

### 2.1. Classification and Phylogenetic Analysis of MYB Subgroup 20 Genes

**Figure 1:**
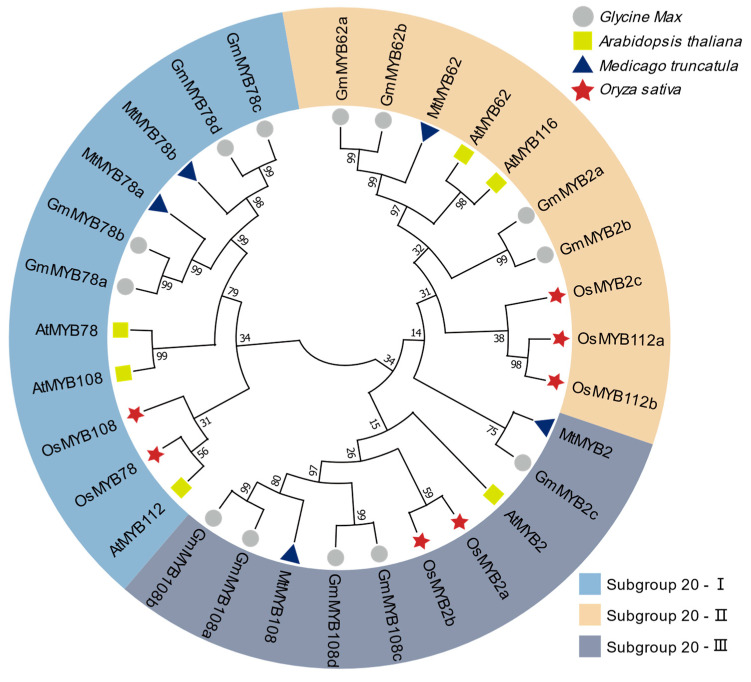
Phylogenetic analysis of MYB-S20 proteins from *Glycine max*, *Arabidopsis thaliana*, *Medicago truncatula*, and *Oryza sativa*. Based on sequences, a neighbor-joining tree constructed using MEGA11 software reveals the evolutionary relationships of S20 members in soybean, Arabidopsis, alfalfa, and rice. Soybean genes are denoted by circles, Arabidopsis genes by squares, Medicago genes by triangles, and rice genes by stars. Color-shaded areas demarcate the three S20 subgroups: Subgroup-I, Subgroup-II, and Subgroup-III. Accession number amino acid sequences for all S20 proteins are detailed in Supplementary Table S1. Bootstrap values = 1000.

The S20 subgroup of MYB transcription factors in *Arabidopsis thaliana* comprise six members: AtMYB2, AtMYB62, AtMYB78, AtMYB108, AtMYB112, and AtMYB116 [15]. Comparative phylogenetic analysis of these genes across soybean (*Glycine max*), alfalfa (*Medicago truncatula*) [25], and rice (*Oryza sativa*) [26] revealed distinct evolutionary trajectories (Figure 1). Based on amino acid sequence alignment, the soybean GmMYB S20 proteins were classified into three subgroups: Subgroup-I GmMYB78s (GmMYB78a, GmMYB78b, GmMYB78c, and GmMYB78d), Subgroup-II GmMYB2s (GmMYB2a, and GmMYB2b), and GmMYB62s (GmMYB62a, and GmMYB62b), and Subgroup-III GmMYB108s (GmMYB108a, GmMYB108b, GmMYB108c, and GmMYB108d), and GmMYB2c (Figure 1, Supplementary Table S1).

In Subgroup 20-I, *MtMYB78a* and *MtMYB78b* cluster within the same clade as *GmMYB78s*, indicating a close evolutionary relationship. These genes are more closely related to *AtMYB78* and *AtMYB108*, but more distantly related to *OsMYB108*, *OsMYB78*, and *AtMYB112*. In Subgroup 20-II, *AtMYB62* and *AtMYB116* are homologous genes and exhibit a close evolutionary relationship with *MtMYB62*, *GmMYB62a*, and *GmMYB62b*. In Subgroup 20-III, *MtMYB108* is highly similar to *GmMYB108s*, but shows a more distant relationship to the rice and Arabidopsis genes (Figure 1). Although *MYB* genes are generally conserved between monocots and dicots, *GmMYB* genes exhibit closer phylogenetic relationships with *M. truncatula*. Notably, soybean harbors a greater number of homologs than *A. thaliana*, *M. truncatula*, or *O. sativa*, which is likely attributable to lineage-specific whole-genome duplication (WGD) events that generated and retained numerous paralogous genes [27].

### 2.2. Chromosomal Mapping of GmMYB S20

**Figure 2:**
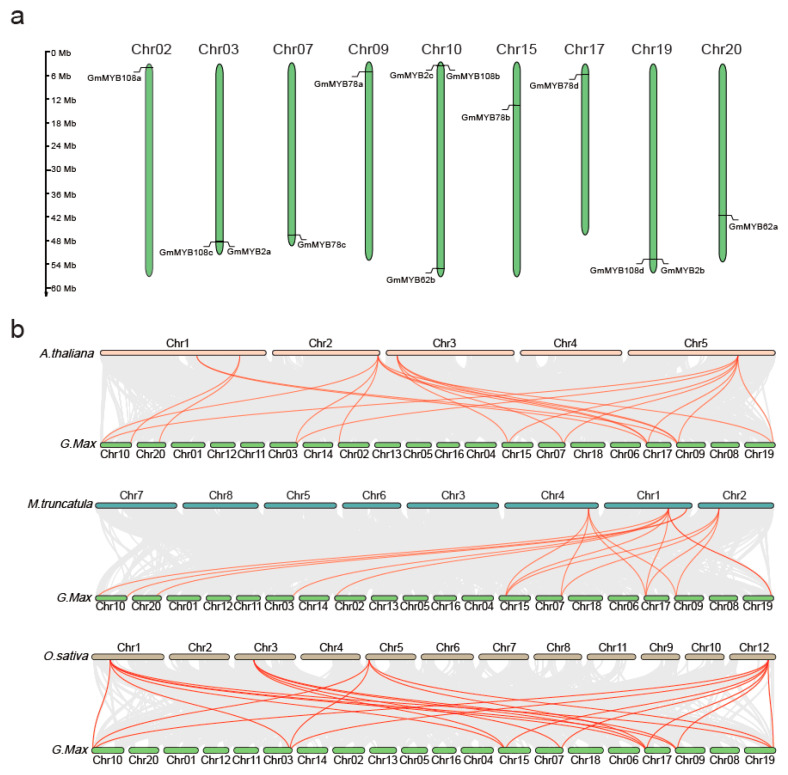
Chromosomal localization of soybean *GmMYB S20* genes and collinearity analysis among species. (**a**) Chromosomal distribution of soybean *GmMYB S20* genes. The diagram was generated using the MapGene2 Chrom web v2 tool, with 13 genes distributed across nine chromosomes. The vertical bars represent soybean chromosomes, and the scale on the left indicates chromosome length. (**b**) Collinearity analysis of MYB S20 among *G. max*, *A. thaliana*, *O. sativa*, and *M. truncatula*. Gray background lines indicate collinear blocks between soybean and other plant genomes, while red lines highlight syntenic *MYB S20* gene pairs.

Chromosomal localization analysis revealed that the 13 *GmMYB* genes are unevenly distributed across nine soybean chromosomes. Notably, several gene pairs are located on the same chromosomes, including *GmMYB108c* and *GmMYB2a* on Chromosome 3, *GmMYB2c* and *GmMYB108b* on Chromosome 10, and *GmMYB2b* and *GmMYB108d* on Chromosome 19. The *GmMYB78s* gene family members are distributed across four different chromosomes: *GmMYB78a* on Chromosome 9, *GmMYB78b* on Chromosome 15, *GmMYB78c* on Chromosome 7, and *GmMYB78d* on Chromosome 17. Additionally, *GmMYB62a* and *GmMYB62b* are located on Chromosomes 20 and 10, respectively (Figure 2a). Collinearity analysis revealed 12 homologous gene pairs in *O. sativa*, 13 in *A. thaliana*, and 16 in *M. truncatula*. Among these, *GmMYB2c* and *GmMYB78s* showed conserved synteny across all four species analyzed (Figure 2b, Supplementary Table S2). Interestingly, orthologs of *MYB62s* were detected in *A. thaliana*, *M. truncatula*, and *G. max*, but were absent in *O. sativa*, suggesting a possible gene loss or functional divergence during evolution (Figure 2b). Duplication events were identified in all three *GmMYB* subgroups, with varying frequencies, supporting the idea of independent evolutionary paths. Overall, these findings underscore a stronger phylogenetic relationship between *GmMYB S20* genes and their *M. truncatula* orthologs, highlighting evolutionary conservation among leguminous species.

### 2.3. Structural and Functional Analysis of GmMYB S20

**Figure 3:**
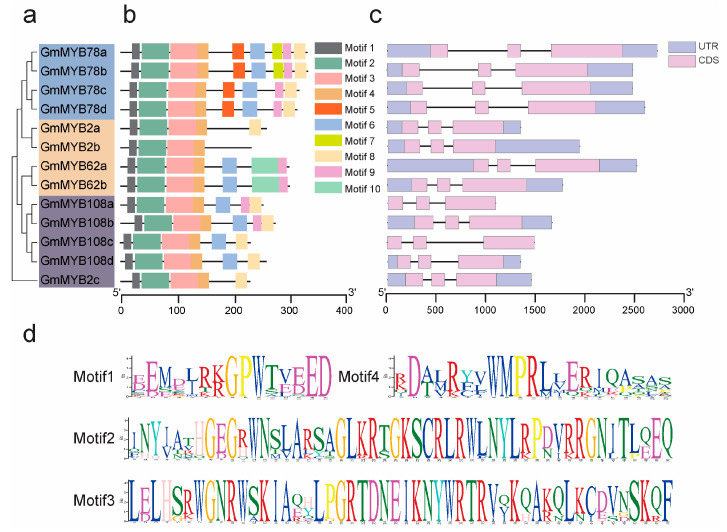
Phylogenetic tree, gene structure, and conserved motifs of GmMYB S20 proteins. (**a**) Phylogenetic tree of GmMYB S20 proteins constructed using the neighbor-joining method. (**b**) Distribution of conserved motifs in GmMYB S20 proteins. Distinct colors represent different motif types. Detailed information on the GmMYB S20 motifs are provided in Supplementary Table S3. (**c**) Gene structure composition of *GmMYB S20* genes, including exons, introns, and untranslated regions (UTRs). Light purple boxes: UTRs; light blue boxes: exons; black lines: introns. The gene structure information of GmMYB S20 is provided in Supplementary Table S4 (**d**). Sequence logos of conserved residues. Conserved motifs in GmMYB S20 amino acid sequences predicted by the MEME Suite. The letter size in motifs 1–4 indicates conservation level at each position.

Based on amino acid sequence similarity, the GmMYB S20 proteins were classified into three distinct subgroups (Subgroup-I, Subgroup-II, and Subgroup-III) (Figure 3a). All members possessed conserved R2R3 domains, which are characteristic of MYB family transcription factors. Using the MEME Suite, we identified ten conserved motifs among the GmMYB S20 proteins. Of these, Motifs 1–4 were consistently present in all subgroup members and thus considered highly conserved (Figure 3b). These four motifs, located at the N-terminus, are responsible for DNA binding activity. However, motifs located in the C-terminal region serve as transcriptional regulatory domains, functioning as either activators or repressors. The motifs organization displayed in Figure 3d correspond to those in Figure 3b, indicating their involvement in DNA-binding activity of GmMYB S20 proteins.

Notably, GmMYB2a, GmMYB2b, and GmMYB2c retained only four to five conserved motifs, which were shared within their subgroups. GmMYB2b uniquely lacked Motif 8, while GmMYB62a and GmMYB62b exclusively harbored Motif 10. Additionally, GmMYB78s specifically contained motif 5, suggesting possible sub-functional divergence among subgroup members (Figure 3b).

To further explore the evolutionary characteristics and functional diversity of *GmMYB S20* genes, we analyzed the exon–intron organization of all 13 members. Despite variations in total gene length, all genes consistently contained three exons. Genes located in close genomic proximity exhibited similar exon–intron structures (Figure 3c), indicating potential functional relatedness.

### 2.4. Cis-Regulatory Elements in GmMYB S20 Promoters

**Figure 4:**
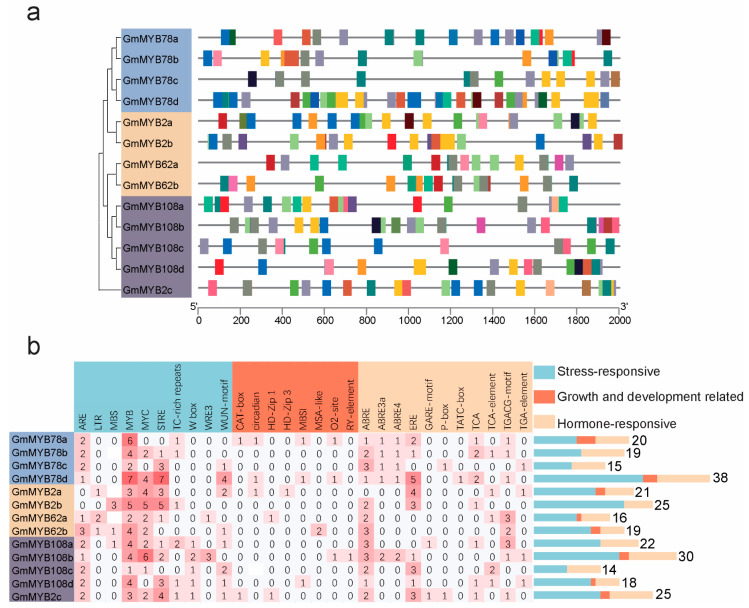
Analysis of *cis*-acting elements in the promoter regions of *GmMYB S20* genes. (**a**) Distribution map of predicted *cis*-acting elements in *GmMYB S20* promoter regions. (**b**) Functional classification and quantitative analysis of *cis*-acting elements. Elements are categorized into three functional groups: stress response, hormone response, and growth/development. A grid heatmap with color bars indicates the quantitative distribution of elements.

*Cis*-acting regulatory element analysis in the promoter regions of the 13 *GmMYB S20* genes identified 283 elements were classified into three functional categories: stress response (168 elements, 60%), hormone response (98 elements, 34%), and growth/development regulation (17 elements, 6%) (Figure 4a, Supplementary Table S5). Stress-responsive motifs included MYB, MYC, and MBS (linked to drought and abiotic stress), ARE (anaerobic induction), TC-rich repeats (plant defense), and WUN (wounding). Growth/development-related motifs included the CAT box, associated with meristem-specific regulation. Among hormone-related elements, ABRE (abscisic acid), TGA-element (auxin), TCA-element (salicylic acid), GARE-motif (gibberellin), and TGACG-motif (jasmonate) were identified [28], with ABRE being the most prevalent (Figure 4b, Supplementary Table S5) [29]. This suggests a critical role for *GmMYB S20* genes in ABA-mediated signaling and abiotic stress responses.

### 2.5. Structural Characterization of GmMYB S20 Proteins

**Figure 5:**
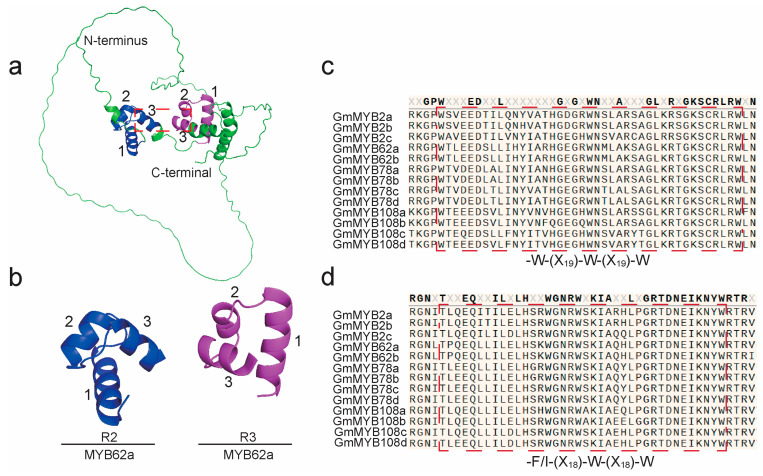
Predicted three-dimensional protein structure of the R2R3-conserved motif in GmMYB S20 protein. (**a**) Predicted three-dimensional structure of the GmMYB62a protein by AlphaFold. The N-terminus indicates the start of the protein synthesis sequence, and the C-terminal indicates the end of the protein synthesis sequence. Blue represents the R2 motif, magenta represents the R3 motif, and green represents the remaining structure. The labels 1, 2, and 3 denote the three alpha-helices within both the R2 and R3 motifs, respectively. (**b**) R2R3 motif of GmMYB62a. The R2R3 motif from (**a**) is magnified, excluding the non-conserved regions (green structure) for closer observation. (**c**,**d**) Conservation analysis of the R2R3 motif sequences across the GmMYB S20 protein. The red dashed boxes highlight the critical regions of the conserved motifs.

The R2R3 domains of GmMYB S20 proteins function as DNA-binding domains (DBDs) [23]. Each domain contains two MYB repeats (R2 and R3), each forming three α-helices that collectively generate a hydrophobic core essential for DNA interaction [30,31]. To examine the structural features of this domain, we selected GmMYB62a as a representative due to its high degree of sequence conservation. The three-dimensional structure of GmMYB62a was predicted using AlphaFold (Figure 5a). The model clearly shows the three α-helices in both R2 (blue) and R3 (magenta) repeats, with non-conserved regions in green (Figure 5a). The N-terminus corresponds to the transcription start site, while the C-terminal corresponds to the transcription termination site. The three α-helices of the R2R3-MYB domain are labeled as 1, 2, and 3, respectively. The third helix of each repeat, which is critical for DNA binding, is highlighted with red dashed boxes (Figure 5a,b). The close spatial arrangement of these helices indicates a cooperative conformation for DNA binding. Secondary structure analysis further confirmed the presence of characteristic motifs: R2 (-W-(X_19_)-W-(X_19_)-W) and R3 (-F/I-(X_18_)-W-(X_18_)-W) (Figure 5c,d), demonstrating high structural conservation in both secondary and tertiary contexts [30,32]. These results suggest that members of the GmMYB S20 subfamily possess a conserved DNA-binding structure, providing a foundation for further investigation into their transcriptional regulatory functions.

### 2.6. GmMYB S20 Genes Expression Patterns

**Figure 6:**
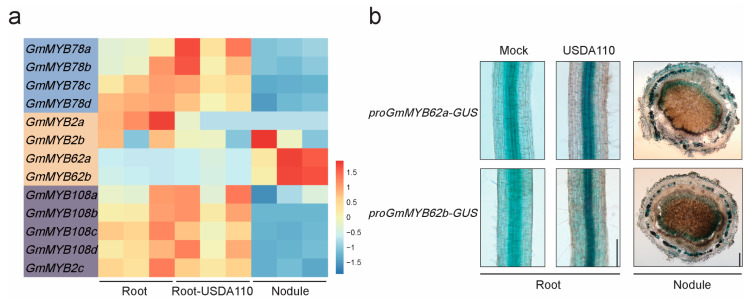
Expression patterns of *GmMYB S20* genes in different tissues. (**a**) Expression levels of *GmMYB S20* genes were determined in the following samples Root, Root-USDA110 (*Bradyrhizobium japonicum* USDA110), Nodule. Differentially expressed genes were identified using thresholds of *p* < 0.05 and |log_2_FC| ≥ 2. Detailed data are provided in Supplementary Table S6. (**b**) Histochemical staining of transgenic roots and nodules expressing *proGmMYB62a: GUS* and *proGmMYB62b: GUS* detected GUS activity in untreated roots, inoculated roots, and nodules at 21 days post inoculation (21 dpi). Scale bar: 100 μm.

Transcriptomic profiling analysis revealed distinct expression patterns of *GmMYB S20* genes in soybean roots and nodules following rhizobial inoculation (Figure 6a, Supplementary Table S6). Most GmMYB S20 family members exhibited little or no change of expression in roots upon inoculation. However, in nodules, *GmMYB62a* and *GmMYB62b* (collectively referred to as *GmMYB62s*) exhibited consistently high basal expression levels, while other family members were largely unexpressed in nodules. Unlike their strong expression in nodules, *GmMYB62s* showed low expression in roots. These results suggest that *GmMYB62s* play a pivotal role in constitutive nodule activity, suggesting their potential involvement in symbiotic regulation. *GmMYB62s* were selected as the focal point for further functional analysis.

To further investigate the expression patterns of *GmMYB62s*, transgenic hairy roots expressing a *proGmMYB62s: GUS* reporter were generated. GUS staining analysis showed that *GmMYB62s* expression increased following rhizobia inoculation, with stronger signals in inoculated root than in untreated roots (Figure 6b). GUS staining of nodules revealed that *proGmMYB62a: GUS* and *proGmMYB62b: GUS* share identical tissue-specific expression patterns, with strong expression observed in the infection zone and sclerenchyma cells of nodules. This expression pattern was consistent with transcriptomic data, reinforcing the hypothesis that GmMYB62s are subject to tissue-specific regulation during nodulation. These findings prompted further investigation into the roles of GmMYB62s in symbiotic signaling and regulation.

### 2.7. GmMYB62s Regulate Nodulation

**Figure 7:**
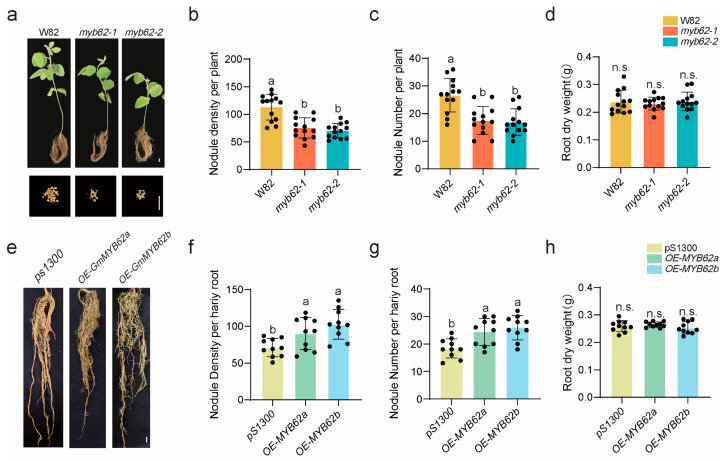
*GmMYB62s* positively regulate nodulation. (**a**) Nodulation phenotypes of wild-type Williams 82 (W82), *myb62-1* and *myb62-2* at 28 dpi. Scale bar = 1 cm. (**b**) Nodule number per plant and nodule dry weight of wild-type W82, *myb62-1* and *myb62-2* at 28 dpi. Error bars represent mean ± SE, n = 13. Different letters indicate statistically significant differences (*p* < 0.05) based on one-way ANOVA followed by Tukey’s multiple comparison test. (**c**) Nodule number per plant of wild-type W82, *myb62-1*, and *myb62-2* after 28 dpi. Error bars represent mean ± SE, n = 13. Different letters indicate statistically significant differences (*p* < 0.05) based on one-way ANOVA followed by Tukey’s multiple comparison test. n = 13. (**d**) Root dry weight per plant (**g**) of wild-type W82, *myb62-1* and *myb62-2*. Error bars represent mean ± SE, n = 13. “n.s.” indicates no significant difference. (**e**) Nodulation phenotypes of empty vector control (pS1300), *OE-GmMYB62a*, and *OE-GmMYB62b* transgenic hairy roots at 28 dpi. Scale bar = 1 cm. (**f**) Nodule density per hair root of empty vector control (pS1300), *OE-GmMYB62a*, and *OE-GmMYB62b* at 28 dpi. Different letters indicate statistically significant differences (*p* < 0.05) based on one-way ANOVA followed by Tukey’s multiple comparison test. (**g**) Nodule number per plant of empty vector control (pS1300), *OE-GmMYB62a*, and *OE-GmMYB62b* at 28 dpi. Error bars represent mean ± SE, n = 10. Different letters indicate statistically significant differences (*p* < 0.05) based on one-way ANOVA followed by Tukey’s multiple comparison test. (**h**) Root dry weight per hair root (**g**) of empty vector control (pS1300), *OE-GmMYB62a*, and *OE-GmMYB62b* transgenic hairy roots at 28 dpi. Error bars represent mean ± SE, n = 10. “n.s.” indicates no significant difference.

To investigate the role of *GmMYB62s* in soybean nodulation, we generated *GmMYB62s* mutants with frameshift mutations or fragment deletions in the *GmMYB62a* and *GmMYB62b* genes using the CRISPR/Cas9 system. GmMYB62a encodes a protein of 307 amino acids, while GmMYB62b encodes a protein of 308 amino acids (Supplementary Table S1). In the *myb62-1* mutant, GmMYB62a exhibits a premature translation termination caused by a nucleotide deletion, resulting in a truncated protein of only 45 amino acids. Similarly, GmMYB62b also undergoes premature termination due to a nucleotide deletion, producing a 21 amino acid polypeptide. In the *myb62-2* mutant, GmMYB62a is truncated to 23 amino acids, and GmMYB62b to 24 amino acids, both due to nucleotide deletions leading to early stop codons. At 28 days post-inoculation (28 dpi) with *Bradyrhizobium japonicum* USDA110, both *myb62-1* and *myb62-2* mutants exhibited significantly reduced root nodule density (Figure 7a,b) and nodule number (Figure 7c) compared to the wild-type Williams 82 (W82), while root dry weight remained unchanged (Figure 7d). These results suggest that *GmMYB62s* act as positive regulators in root nodule formation. To further elucidate the role of *GmMYB62s* in nodulation, *GmMYB62a* and *GmMYB62b* were overexpressed in hairy roots. At 28 dpi, the nodule density (Figure 7e,f) and number (Figure 7g) in overexpression lines was significantly increased than in the empty vector control (pS1300), with no differences in root dry weight (Figure 7h). Collectively, these findings demonstrate that GmMYB62s play a positive regulatory role in root nodule formation in soybean.

### 2.8. Transcriptomic Analysis of GmMYB62s Reveals Functional Pathways

To further investigate how *GmMYB62s* regulate root nodulation, we performed RNA-Seq analysis on W82 and *myb62-1* roots at 7 dpi with *B. japonicum* USDA110. Each genotype was analyzed using three biological replicates. DEGs were identified using the criteria of *p* < 0.05 and |log_2_FC| ≥ 2.

**Figure 8:**
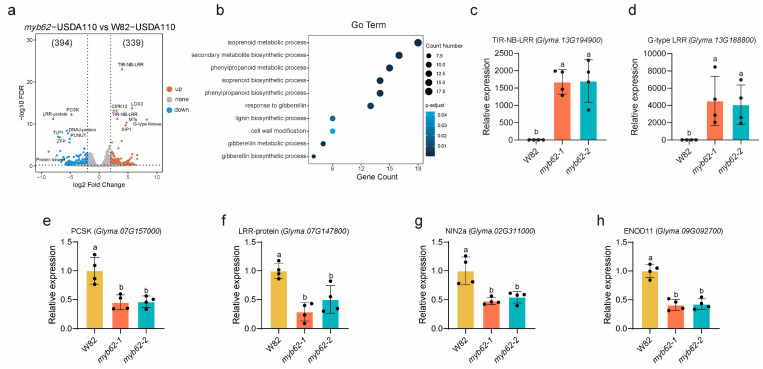
RNA-Seq analysis of W82 and *myb62-1*, and RT-qPCR validation of related gene expression. (**a**) Volcano plot of differentially expressed genes (DEGs) in *myb62-1* vs. W82 roots under *B. japonicum* USDA110 inoculation 7dpi. Red: 399 significantly upregulated genes; blue: 394 significantly downregulated genes. (**b**) Significantly enriched Go terms for DEGs identified in W82 vs. *myb62-1* under *B. japonicum* USDA110 inoculation. The vertical axis displays Go term names, while the horizontal axis represents the Rich Factor, a higher value indicates greater enrichment significance. The size of the data points corresponds to the number of genes associated with the term, and the color corresponds to the range of adjusted *p*-values (*p*-adjust). (**c**–**h**) Expression of selected genes was assessed by RT-qPCR in root samples collected 7 days post-inoculation (dpi) with rhizobia from wild-type W82 and *myb62-1*. (**c**,**d**) Validation of RNA-Seq-upregulated genes: *TIR-NB-LRR* (*Glyma.13G194900*) and *G-type leucine-rich repeat* (*G-type LRR*, *Glyma.13G188800*). (**e**,**f**) Validation of RNA-Seq-downregulated genes: *Proprotein Convertase Subtilisin/Kexin* (*PCSK*) and *leucine-rich repeat protein* (*LRR protein*). (**g**,**h**) Expression of symbiosis marker genes *Nodule inception 2* (*NIN2*, *Glyma.02G311000*) and *Early Nodulin 11* (*ENOD11*, *Glyma.09G092700*). *GmActin* was used as the reference gene for normalization. Data represent means ± SD from four biological replicates. Different letters indicate statistically significant differences (*p* < 0.05) determined by one-way ANOVA with multiple comparisons.

A total of 733 DEGs were identified, including 339 upregulated and 394 downregulated genes in the *myb62-1* mutant compared to W82 (Figure 8a). Gene Ontology (GO) enrichment analysis revealed significant associations across 10 biological pathways (Figure 8b). Gene Ontology (GO) enrichment analysis revealed significant enrichment in the “isoprenoid biosynthetic process” (GO:0008299, 14 genes) and “terpenoid metabolic process” (GO:0006720, 18 genes) (Figure 8b). These two pathways contribute to the synthesis of terpenoid compounds via the mevalonate (MVA) and methylerythritol phosphate (MEP) pathways, which are involved in photosynthesis, defense, and developmental regulation [33]. Additionally, genes associated with “gibberellin biosynthesis” (GO:0009686, seven genes), “gibberellin metabolism” (GO:0009685, eight genes), and “gibberellin response” (GO:0009739, 13 genes) were also enriched. Gibberellins are known to facilitate rhizobial infection by promoting root hair deformation and infection thread formation during early nodulation, and subsequently activate the autoregulation of nodulation (AON) pathway to suppress excessive nodulation [34,35]. Additional enrichment was observed for genes associated with “cell wall modification” (GO:0042545, nine genes), “phenylpropanoid biosynthetic process” (GO:0009699, 14 genes), “lignin biosynthesis” (GO:0009809, nine genes) (Figure 8b, Supplementary Table S7). Given the importance of cell wall flexibility for successful rhizobial colonization, the upregulation of these pathways in *myb62-1* may increase cell wall rigidity and activate defense barriers [36]. Notably, the phenylpropanoid and lignin pathways contribute to flavonoid and lignin biosynthesis, both of which modulate cell wall structure and defense responses [37,38].

Among the highly expressed genes with a significant −log_10_FDR, we identified two TIR-NB-LRR genes (*Glyma.13G194900*, *Glyma.13G190800*) and one G-type LRR gene (*Glyma.13G188800*), which are known to play essential roles in plant immunity [39,40] (Figure 8c,d). Additionally, the *LOX* gene (*Glyma.04G105900*), encoding linoleate 9-lipoxygenase, is involved in defense responses induced by chitosan and produces reactive molecules that can directly inhibit pathogens or activate immune-related genes [41]. A methyltransferase gene (*Glyma.03G045400*) was also upregulated, which is critical for chitosan-induced defense responses, was also among the DEGs. Although functional roles of some downregulated genes, we validated two significantly downregulated targets: *Proprotein Convertase Subtilisin/Kexin* (*PCSK*) and *Leucine-Rich Repeat Protein* (*LRR protein*). Their expression trends by RT-qPCR aligned with RNA-Seq data (Figure 8e,f). We further validated the expression of symbiosis marker genes *Nodule inception 2* (*NIN2*) [42] and *Early Nodulin 11* (*ENOD11*) [43], observing reduced levels in the *myb62-1* mutant (Figure 8g,h). These results suggest that *GmMYB62s* may fine-tune the expression levels of immunity-related genes to maintain immune homeostasis in the rhizosphere, preventing excessive defense responses that may hinder symbiosis (Figure 8, Supplementary Table S8).

Taken together with phenotypic data, these findings suggest that *GmMYB62s* may promote rhizobial colonization by repressing the biosynthesis of phenylpropanoids or lignin in the secondary metabolic pathways, thereby reducing cell wall rigidity. In the absence of *GmMYB62s*, de-repression of these pathways may lead to the overaccumulation of defense-related metabolites, increased cell wall rigidity, and carbon–nitrogen imbalances, ultimately resulting in reduced nodulation, stunted plant growth, and chlorosis.

## 3. Discussion

This study reveals that *GmMYB62s*, members of the *GmMYB S20* genes, act as key transcription factors regulating symbiotic nodulation in soybean (Figure 7a–h). Upon rhizobial inoculation, *GmMYB62s* appear to promote nodulation by fine-tuning immune thresholds, enhancing cell wall plasticity, and coordinating with gibberellin signaling (Figure 8a,b). These findings expand our understanding of MYB-mediated regulation in legume symbiosis and offer valuable molecular insights into the mechanisms governing nodulation efficiency.

Recent studies have further underscored the functional diversity of MYB transcription factors across legumes. In *Medicago sativa* (alfalfa), the MsMYB206-MsMYB450-MsHY5 transcription factor complex enhances antioxidant capacity under salt stress by regulating flavonoid biosynthetic gene expression [44]. A genome-wide identification of the R2R3-MYB family in *Trifolium pratense* (red clover) identified candidate genes potentially regulating isoflavonoid biosynthesis [45]. In *Lotus japonicus*, LjMYB15 regulates plant defense metabolism by activating isoflavonoid synthesis genes in response to UV-B radiation, while LjMYB13 confers salt stress tolerance through mechanisms involving Cl^−^ homeostasis, root architecture remodeling, and vestitol-mediated oxidative protection [46]. Furthermore, genome-wide analysis of 1R-MYB genes in *Trifolium repens* L. (white clover) identified five genes (*TrMYB41*, *TrMYB49*, *TrMYB94*, *TrMYB125*, *TrMYB130*) responsive to drought stress [47]. While these findings highlight the crucial roles of MYB transcription factors in diverse stress responses and specialized metabolism in legumes, direct evidence linking specific MYB factors to the process of symbiotic nodulation has remained limited. Our work on GmMYB62s addresses this gap by demonstrating their essential and specific function in positively regulating nodule formation and symbiotic efficiency.

The expansion of the *GmMYB S20* subfamily in soybean is likely a result of WGD events. Phylogenetic analysis showed that *GmMYB62s* are more closely related to their legume orthologs than to those in Arabidopsis or rice homologs, suggesting conserved roles within leguminous species (Figure 1). The high conservation of R2R3 domains, combined with subgroup specific motifs such as motif 10 in *GmMYB62s* and motif 5 in *GmMYB78s* (Figure 3a), may underlie their functional specialization in balancing symbiosis and stress responses. Furthermore, the gene structure analysis showed consistent exon–intron organization among close paralogs (Figure 3b), implying shared evolutionary origins. However, variation in non-conserved regions and motif compositions across subgroups suggests sub-functionalization or neofunctionalization following gene duplication (Figure 3b). These structural divergences may allow *GmMYB62s* to integrate diverse signaling inputs, such as hormone pathways and environmental cues, to mediate context-dependent transcriptional responses. Taken together, the evolutionary trajectory of the *GmMYB S20* family illustrates a balance between conservation and diversification that may have endowed legumes with specialized regulatory mechanisms for symbiosis.

GO enrichment analysis revealed that *GmMYB62*-regulated gene sets are significantly enriched in gibberellin biosynthetic and signaling pathways, reinforcing their proposed role in nodulation control [48] (Figure 8b). Gibberellins are known to support early rhizobial infection processes and activate the autoregulation of nodulation (AON) pathway to prevent excess nodules [35]. In parallel, GmMYB62s may fine-tune immune responses by repressing the expression of defense-related genes and secondary metabolites such as lignin and phenylpropanoids, which can inhibit rhizobial colonization when over accumulated. Compared to wild-type W82, RT-qPCR analysis revealed significantly reduced expression levels of the symbiotic nodulation genes *GmNINs* and *GmENOD11* in the *myb62-1* mutant. This finding suggests that MYB62a and MYB62b promote symbiotic nodulation, likely through the regulation of *GmNINs* and *GmENOD11* (Figure 8g,h). By softening the cell wall and maintaining immune homeostasis, GmMYB62s create a permissive environment for symbiosis. These findings align with previous studies in Arabidopsis, where MYB factors orchestrate secondary metabolism to balance growth and immunity [15]. The function of *GmMYB62s* in promoting nodulation presents opportunities for legume crop breeding. Overexpression of *GmMYB62s* promotes nodulation. Furthermore, *GmMYB62s* knockout lines display chlorosis and reduced shoot biomass (Figure 7a), which may be due to impaired phosphate uptake or nitrogen fixation deficiency. Therefore, it is necessary to further analyze the shoot phenotype using stable overexpression transgenic lines. Future efforts could focus on engineering *GmMYB62s* alleles to achieve optimized nodulation efficiency under variable environmental conditions. Additionally, the conserved collinearity between *GmMYB62s* and their homologs in *Medicago truncatula* underscores the potential for translating this regulatory module across legume species.

Although this study delineates the transcriptional regulatory landscape of *GmMYB62s* in nodulation, further validation is required to identify their direct target genes and upstream regulators. Future work will involve chromatin immunoprecipitation followed by sequencing (ChIP-seq) to pinpoint promoter regions bound by *GmMYB62s*, as well as protein–protein interaction assays to explore regulatory complexes. Additionally, the roles of other members within the GmMYB S20 subfamily merit investigation to build a more comprehensive picture of MYB-mediated symbiotic control. Ultimately, dissecting the regulatory network governed by *GmMYB S20* genes will deepen our understanding of legume–rhizobia interactions and support the development of crops with improved nitrogen-fixation capacity.

## 4. Materials and Methods

### 4.1. Identification and Phylogenetic Analysis of GmMYB S20 Genes

Protein sequences of *Arabidopsis thaliana* MYB S20 members were retrieved from the published literature [15]. Homologous proteins in *Glycine max* (Wm82.a6v1), *Medicago truncatula* (Mt.4.0v1), and *Oryza sativa* (Os.v7.0) were identified via BLASTP searches against annotated proteomes available in the Phytozome database (https://phytozome-next.jgi.doe.gov/, accessed on 5 May 2025) using a stringent E-value cutoff of 1 × 10^−10^. Multiple sequence alignment was performed, and a phylogenetic tree was constructed using the neighbor-joining (NJ) method in MEGA11 [49]. Tree reliability was assessed using 1000 bootstrap replicates (Supplementary Table S1).

### 4.2. Chromosomal Localization

Chromosomal positions of *GmMYB S20* genes were extracted from the Phytozome database. Physical positions, chromosomal coordinates, and gene lengths were visualized using MapGene2Chrom (http://mg2c.iask.in/mg2c_v2.0/, accessed on 15 April 2025) to generate a chromosomal distribution map.

### 4.3. Collinearity Analysis

To assess genomic conservation and collinearity, syntenic relationships of *GmMYB S20* genes were analyzed among *A. thaliana*, *G. max*, *M. truncatula*, and *O. sativa* using the One Step MCScanX and Dual Synteny Plot tools in TBtools v2.313 [50]. The results were visualized to illustrate interspecies gene conservation and potential genomic rearrangements (Supplementary Table S2).

### 4.4. Gene Structure and Conserved Motif Analysis

Conserved motifs were identified using the MEME Suite (https://meme-suite.org/meme/tools/meme, accessed on 18 April 2025) with default parameters [51] (Supplementary Table S3). Exon–intron structures of *GmMYB S20* genes were visualized using TBtools based on genomic and coding sequence (CDS) data [50,52] (Supplementary Table S4). R2R3-MYB domains were annotated and graphically represented using SnapGene software v6.0.2.

### 4.5. Prediction of Cis-Regulatory Elements

Promoter sequences (2000 bp upstream of the start codon) of *GmMYB S20* genes were retrieved from the Phytozome database (Supplementary Table S5). *Cis*-regulatory elements were identified using the PlantCARE tool (https://bioinformatics.psb.ugent.be/webtools/plantcare/html/) (accessed on 5 May 2025) [53]. Distribution of these elements was visualized with TBtools to predict regulatory functions [50].

### 4.6. Three-Dimensional Protein Structure Modeling

The three-dimensional structures of GmMYB S20 proteins were predicted using the AlphaFold database (https://alphafold.ebi.ac.uk/) (accessed on 12 May 2025). Protein models were visualized and annotated using PyMOL to highlight conserved domains and structural features.

### 4.7. RNA-Seq Analysis and Functional Enrichment

For Figure 6a RNA-seq data, roots of the wild-type Williams 82 (W82) treated with Bradyrhizobium japonicum USDA110 for 7 days, along with water-treated roots as the control, were harvested. The “Nodule” samples represent specifically the nodules developed after treatment with *Bradyrhizobium japonicum* USDA110 for 28 days (on W82 plants). Only nodules were collected, and 2g of nodule material was used as the sample. Root nodules of wild-type W82 and *myb62-1* and *myb62-2* were treated with *Bradyrhizobium japonicum* USDA110 for 7 days. Three biological replicates of each W82, *myb62-1*, and *myb62-2*, each consisting of 2g of material, were collected. Total RNA was extracted from these samples using the Vazyme RNA extraction kit. The resulting RNA samples were then sent to BGI (Huada Gene) for RNA sequencing (RNA-seq). Our RNA-seq data has been deposited in the National Genomics Data Center (NGDC) Genome Sequence Archive (GSA) under accession number CRA027643. Data analysis was performed using the bioinformatics analysis platform provided by BGI (https://biosys.bgi.com/#/loading/bgi/) (accessed on 15 May 2025). DEGs were identified using the DESeq2 package, with thresholds set at |log2(fold change)| ≥ 2 and *p*-value < 0.05 [54]. To validate the results, the DEGseq package was also utilized, applying FDR (false discovery rate) cutoff of <0.001. Gene Ontology (GO) enrichment analysis of significant DEGs was conducted using the SoyMD online platform (https://yanglab.hzau.edu.cn/SoyMD/#/tools/go) (accessed on 16 May 2025), which provides integrated GO annotations for *Glycine max* (Supplementary Table S8). Enriched biological processes, molecular functions, and cellular components were visualized using R with the ggplot2 package to generate pathway-specific heatmaps and bar plots [55]. The RNA sequencing data generated in this study have been deposited.

### 4.8. Vector Construction

For overexpression constructs, the *pS1300* vector was digested with HindIII and SalI restriction enzymes. The full-length cDNA sequences of *GmMYB62a* and *GmMYB62b* were inserted into the linearized vector using a homologous recombination approach, performed with a seamless cloning enzyme mix at 50 °C for 15 min. Recombinant plasmids were transformed into Escherichia coli DH5α competent cells, and positive colonies were confirmed by Sanger sequencing. Verified constructs were then introduced into Agrobacterium tumefaciens strain K599 for subsequent plant transformation.

For CRISPR/Cas9 knockout constructs, sgRNA sequences targeting *GmMYB62a* and *GmMYB62b* were synthesized and directionally cloned into the pGES401 vector (clustered regularly interspaced short palindromic repeats-associated systems gene editing vector) via Golden Gate assembly [56]. sgRNA expression cassettes were assembled using BsaI restriction sites to enable precise modular integration. The resulting constructs were first transformed into the *Escherichia coli* sequence verified, and then transferred into Agrobacterium for downstream genetic transformation. The primers were listed in Supplementary Table S9.

### 4.9. Plant Materials and Growth Conditions

*G. max* cultivar W82 was used for all experiments. Seeds were surface-sterilized and germinated on autoclaved vermiculite in a controlled growth chamber set at 26 °C with a 16 h light/8 h dark photoperiod. For rhizobial inoculation assays, seedlings were grown in sterilized vermiculite for 7 days and subsequently inoculated with *Bradyrhizobium japonicum* USDA110 (OD_600_ = 0.05). Phenotypic assessments, including nodulation efficiency and root architecture analysis, were performed at 28 dpi (days post-inoculation).

To generate *GmMYB62s* knockout mutants, CRISPR/Cas9 gene editing was employed. Three sgRNAs (single-guide RNAs) targeting distinct exonic regions of *GmMYB62a* and *GmMYB62b* were designed using the Sequence Scan for CRISPR (http://crispr.dfci.harvard.edu/SSC/) (accessed on 15 May 2025) [57]. The sgRNA expression cassettes were assembled into the pGES401 vector via Golden Gate cloning [56]. The resulting constructs were introduced into *Agrobacterium tumefaciens strain* K599 for transformation into soybean. Transgenic lines were screened for homozygous mutations by PCR amplification and Sanger sequencing, followed by phenotypic evaluation under standardized rhizobial symbiosis conditions.

### 4.10. Nodule Biomass Measurement

Nodules were harvested at 28 dpi, surface-dried, and oven-dried at 60 °C to constant weight. Nodule number and total dry weight per plant were measured. Average nodule weight was calculated by dividing total dry weight by nodule number.

### 4.11. GUS Histochemical Staining

Hairy roots inoculated with *B. japonicum* for 21 days were stained with X-Gluc solution (50 mM NaH_2_PO_4_, 50 mM Na_2_HPO_4_, 0.5 mM K_3_Fe(CN)_6_, 0.5 mM K_4_Fe(CN)_6_, 10 mM EDTA, 0.1% Triton X-100, 2 mM X-Gluc) at 37 °C for 6–8 h. GUS staining was visualized under an Olympus BX53 microscope(Olympus Corporation, Tokyo, Japan). For cross-sectioning, nodules were embedded in low-melting-point agarose (Coolaber, Beijing, China, Cat#CA1351) and sectioned at 50 μm using a Leica VT1000S vibratome [58].

### 4.12. Hairy Root Transformation

Plump seeds were germinated in vermiculite. After 4 days, hypocotyls were excised using sterile scissors, and *Agrobacterium rhizogenes* strain K599 harboring *35S:MYB62a* or *35S:MYB62b* constructs was applied to the wound sites. The seedlings were then placed on Petri dishes containing water-saturated filter paper and co-cultivated overnight in darkness. The following day, seedlings were transplanted into pots filled with moist vermiculite, and any adventitious roots that developed on the hypocotyls were carefully removed [59].

### 4.13. Agrobacterium-Mediated Transformation of Soybean

Mature soybean seeds were surface-sterilized using chlorine gas (generated from commercial bleach and HCl) and germinated on GM medium or hydrated in darkness. Agrobacterium tumefaciens EHA101 cultures were grown in YEP medium to OD_650_ = 0.6, pelleted, and resuspended in liquid CM medium supplemented with 0.2 mM acetosyringone (AS). Cotyledonary node explants excised from germinated seeds were infected with the Agrobacterium suspension for 20–30 min, co-cultivated on CM solid medium (23 °C, darkness, 3–5 days), and transferred to shoot induction (SI) medium containing glufosinate or glyphosate as selection agents. Explants were cultured under light (24 °C, 18-h photoperiod) with biweekly subculturing onto fresh SI medium for 4 weeks. Developing shoots were then moved to shoot elongation (SE) medium with selection agents and subcultured every 2 weeks. Elongated shoots (>3 cm) were excised, dipped in 1 mg/mL indole-3-butyric acid (IBA), and rooted on RM medium. Rooted plantlets were acclimatized in soil under controlled conditions [60].

### 4.14. RNA Extraction and RT-qPCR Analysis

Root samples from wild-type W82, *myb62-1*, and *myb62-2* plants, which were inoculated with *B.japonicum* USDA110 for 7 days, were collected. Total RNA was extracted using the Vazyme Total RNA Extraction Kit (Vazyme, Nanjing, China). Genomic DNA contamination was eliminated with Vazyme 4× gDNA Wiper Mix, followed by cDNA synthesis using Vazyme 5× Hiscript^®^ qRT SuperMix II. RT-qPCR was performed with ABclonal 2× Universal SYBR Green Fast qPCR Mix on a Bio-Rad CFX96 Real-Time PCR Detection System. Four biological replicates were analyzed per sample. *GmActin* served as the reference gene for expression normalization [61]. The primers were listed in Supplementary Table S9.

## 5. Conclusions

This study lays the foundation for GmMYB research by comprehensively resolving the evolutionary dynamics of the GmMYB S20 subfamily, including chromosomal distribution, motif diversification, and legume-specific conservation and deciphering the functional role of GmMYB62s in symbiosis. We demonstrate that GmMYB62s act as master regulators of nodulation, where CRISPR/Cas9 knockout impairs nodule formation while overexpression enhances symbiotic efficiency. Mechanistically, GmMYB62s balance immune thresholds, fine-tune gibberellin signaling, and remodel cell wall plasticity via repression of lignin/phenylpropanoid biosynthesis to facilitate rhizobial colonization. These insights provide a theoretical framework for soybean symbiosis research and establish GmMYB62s as promising targets for molecular breeding of elite soybean cultivars with optimized nitrogen-fixing capacity.

## Data Availability

Data are contained within the article and Supplementary Materials.

## Supplementary Materials

The following supporting information can be downloaded at https://www.mdpi.com/article/10.3390/plants14142240/s1, Table S1: List of the MYB S20 proteins from *Glycine max*, *Medicago truncatula*, *Arabidopsis thaliana*, and *Oryza sativa*; Table S2: List of *GmMYB S20* homologous gene pairs identified in *Glycine max*, *Medicago truncatula*, *Arabidopsis thaliana*, and *Oryza sativa*; Table S3: The conserved motifs of GmMYB S20 proteins; Table S4: The gene structure composition of GmMYB S20 genes. Table S5: The promoter *cis*-acting elements of *GmMYB S20* genes; Table S6: Expression Levels of *GmMYB S20* Genes from RNA-Seq Analysis; Table S7: DEGs selected via volcano plot; Table S8: Functionally enriched GO pathways; Table S9: Primers used in this study.
